# Mutational Profiles Reveal an Aberrant TGF-β-CEA Regulated Pathway in Colon Adenomas

**DOI:** 10.1371/journal.pone.0153933

**Published:** 2016-04-21

**Authors:** Jian Chen, Gottumukkala S. Raju, Wilma Jogunoori, Vipin Menon, Avijit Majumdar, Jiun-Sheng Chen, Young Jin Gi, Yun Seong Jeong, Liem Phan, Mitchell Belkin, Shoujun Gu, Suchin Kundra, Nipun A. Mistry, Jianping Zhang, Xiaoping Su, Shulin Li, Sue-Hwa Lin, Milind Javle, John S. McMurray, Thomas F. Rahlfs, Bibhuti Mishra, Jon White, Asif Rashid, Nicole Beauchemin, Brian R. Weston, Mehnaz A. Shafi, John R. Stroehlein, Marta Davila, Rehan Akbani, John N. Weinstein, Xifeng Wu, Lopa Mishra

**Affiliations:** 1 Department of Gastroenterology, Hepatology and Nutrition, The University of Texas MD Anderson Cancer Center, Houston, Texas, United States of America; 2 Institute of Clinical Research, Veterans Affairs Medical Center, Washington DC, United States of America; 3 Departments of Molecular and Cellular Oncology, The University of Texas MD Anderson Cancer Center, Houston, Texas, United States of America; 4 Center for Translational Medicine, Department of Surgery and George Washington Cancer Center, George Washington University, Washington DC, United States of America; 5 Departments of Bioinformatics and Computational Biology, The University of Texas MD Anderson Cancer Center, Houston, Texas, United States of America; 6 Department of Pediatrics, The University of Texas MD Anderson Cancer Center, Houston, Texas, United States of America; 7 Department of Translational Molecular Pathology, The University of Texas MD Anderson Cancer Center, Houston, Texas, United States of America; 8 Departments of Gastrointestinal Medical Oncology, The University of Texas MD Anderson Cancer Center, Houston, Texas, United States of America; 9 Department of Experimental Therapeutics, The University of Texas MD Anderson Cancer Center, Houston, Texas, United States of America; 10 Department of Anesthesiology and Perioperative Medicine, The University of Texas MD Anderson Cancer Center, Houston, Texas, United States of America; 11 Department of Pathology, The University of Texas MD Anderson Cancer Center, Houston, Texas, United States of America; 12 Goodman Cancer Research Centre and Departments of Biochemistry, Medicine and Oncology, McGill University, Montreal, Canada; 13 Department of Epidemiology, The University of Texas MD Anderson Cancer Center, Houston, Texas, United States of America; Baylor University Medical Center, UNITED STATES

## Abstract

Mutational processes and signatures that drive early tumorigenesis are centrally important for early cancer prevention. Yet, to date, biomarkers and risk factors for polyps (adenomas) that inordinately and rapidly develop into colon cancer remain poorly defined. Here, we describe surprisingly high mutational profiles through whole-genome sequence (WGS) analysis in 2 of 4 pairs of benign colorectal adenoma tissue samples. Unsupervised hierarchical clustered transcriptomic analysis of a further 7 pairs of adenomas reveals distinct mutational signatures regardless of adenoma size. Transitional single nucleotide substitutions of C:G>T:A predominate in the adenoma mutational spectrum. Strikingly, we observe mutations in the TGF-β pathway and CEA-associated genes in 4 out of 11 adenomas, overlapping with the Wnt pathway. Immunohistochemical labeling reveals a nearly 5-fold increase in CEA levels in 23% of adenoma samples with a concomitant loss of TGF-β signaling. We also define a functional role by which the CEA B3 domain interacts with TGFBR1, potentially inactivating the tumor suppressor function of TGF-β signaling. Our study uncovers diverse mutational processes underlying the transition from early adenoma to cancer. This has broad implications for biomarker-driven targeting of CEA/TGF-β in high-risk adenomas and may lead to early detection of aggressive adenoma to CRC progression.

## Introduction

Advanced colorectal cancer (CRC) remains the third most common cancer in both men and women, with approximately 1.3 million new CRC cases diagnosed in the world every year [1]. Late-stage disease continues to have a dismal survival rate, with over 45% of patients dying of recurrence despite adjuvant therapy [2]. Early detection, prevention, and screening have been central to reducing mortality rates [3–5]. However, these processes are limited by variations among subjects, variability in the sensitivity of the screening procedure, and lack of reliable methods of identification of early high-risk patients. High risk adenomas are conventionally defined clinically as those that are larger than 1 cm. These adenomas are considered to give rise to overt carcinomas and are an important criterion in colonoscopy and screening studies [3, 4, 6, 7]. Moreover, while sequence analyses demonstrating increased numbers of somatic mutations correlate with increased cancer risk have been well documented in many cancers, these have yet to be clearly described for colon adenomas [8–11]. In addition, the driving pathways in CRC have been defined to a large (though not complete) extent- the *APC-Wnt* and TGF-β pathways have been established as modulators of GI stem cells and drivers of CRC. Thus mutations in members of these pathways would carry further significance [12]. It is becoming increasingly clear both that CRC can be missed because it may occur rapidly and that other risk factors and mutational processes may be involved [6].

Several critical drivers and pathways important for the initiation and progression of CRC have been identified and investigated extensively [12–15]. These include the WNT, TGF-β, RAS-MAPK, PI3K, P53, and DNA mismatch-repair pathways. Still, limited insight exists into the determinants of early colorectal neoplasia, including the full spectrum of molecular drivers involved in colorectal neoplastic initiation and pre-invasive progression. In addition, mutational processes and signatures remain poorly characterized in adenomas which could lead to CRC. We hypothesized that defining such mutational signatures could identify rapid progressors to overt CRC as well as new targets for CRC prevention.

In the present study, we conducted whole-genome sequencing on 4 pairs of adenoma samples and whole-transcriptome RNA sequencing on 7 matched colon adenoma samples and normal mucosa. Using unsupervised hierarchical clustering, we observed distinct mutational signatures in the TGF-β pathway and CEA-associated genes, overlapping with the Wnt pathway. Critically, we find a marked increase of CEA levels (nearly 5-fold) in 23% of adenoma samples with commensurate loss of TGF-β signaling and provide mechanistic insight of CEA-mediated TGF-β signaling. Our findings may lead to early detection of aggressive adenoma-CRC progression. Furthermore, these findings have broad implications for cancer prevention, particularly in view of the availability of a CEA vaccine that may be amenable to a high-risk population.

## Results

**Fig 1:**
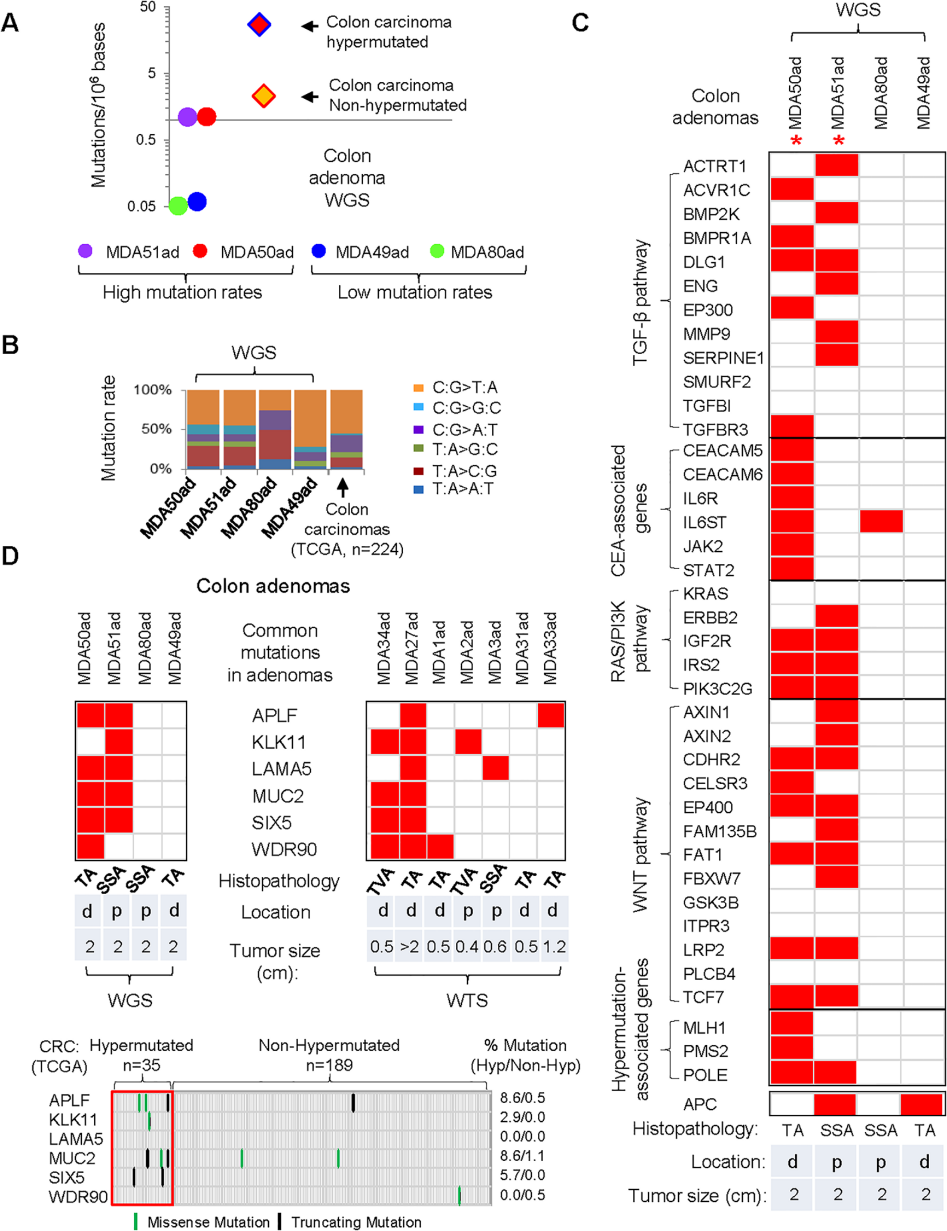
Frequent mutations are observed in colon adenomas. (A) Mutation frequency detected by whole-genome (WGS) sequencing of colon adenoma tissues. The dot represents the number of mutations per Mb in one adenoma sample. The red and orange diamonds represent the median mutation per Mb as observed in the colorectal cancer samples from the TCGA database. (B) Transitional single nucleotide substitutions of C:G > T:A predominate in the adenoma mutational spectrum. (C) Mutation profile of 4 pairs of matched colon adenoma samples and normal mucosa. Each colored dot indicates a mutation in the sample. *: adenomas with high mutation rates. TA: Tubular adenoma; TVA: Tubulovillous adenoma; SSA: Sessile serrated adenoma. Location d: distal; p: proximal. (D) Six most commonly mutated genes observed in 11 colon adenoma tissues (upper panels). These commonly mutated genes in adenoma samples are not frequently mutated in the hypermutated (n = 35, 0–8.6%) and non-hypermutated (n = 189, 0–1.1%) TCGA CRC carcinoma cohort (total n = 224, lower panel).

Genome-wide analysis enables predictive modeling of genetic pathways which drive many cancers, genetic diseases, and human syndromes [16, 17]. To gain a comprehensive understanding of the mutational landscape of genetic alternations taking place in adenomas, whole-genome sequencing (WGS) analysis for 4 adenoma samples was carried out, with a further whole-transcriptome sequencing (WTS) of 7 pairs of matched colon adenoma samples and normal mucosa (S1 Fig and S1 Table). Through the analyses of WGS and WTS data, we detected 7358 non-synonymous somatic mutations in 4610 genes. The WGS shows 1709 average mutations after normalization with an average mutation frequency of 0.55 mutations per 10^6^ bases. Aberrant mutational profiles are detected with distinct mutational signatures among the WGS samples: two samples with high mutation rates (>1.0 mutations/10^6^ bases): MDA50ad-TA and MDA51ad-SSA (Fig 1A); two with low mutation rates: MDA49ad-TA and MDA80ad-SSA (Fig 1A). Compared with colon carcinomas in The Cancer Genome Atlas (TCGA) cohort, the two samples with the highest mutations we screened have similar rates to that of non-hypermutated samples in TCGA colon carcinomas [12]. Even though the mutation rates detected by RNA sequencing may be affected by RNA editing, gene transcripts and tumor heterogeneity [18], the WTS indicate 74 average mutations after normalization with an average mutation frequency of 1.02 mutations per 10^6^ bases (S1 Table). The WTS samples also indicate aberrant mutational profiles: MDA27ad-TA and MDA34ad-TVA with high mutation rates; MDA1ad-TA, MDA2ad-TVA, MDA3ad-SSA and MDA33ad-TA with intermediate mutation rates; MDA31ad-TA with low mutation rates (S2A Fig). Furthermore, we find that transitional single nucleotide substitutions of C:G > T:A predominate in both adenoma mutational spectrums and TCGA colon carcinomas (Fig 1B and S2B Fig).

CRC TCGA studies have validated that frequent mutations of TGF-β signaling are observed in over 80% of proximal colon carcinomas [12]. To explore major affected pathways, we analyzed for alterations in multiple pathways implicated in colon cancer (Fig 1C). Two adenomas, 1 sessile serrated adenoma MDA51ad-SSA and 1 tubular adenoma MDA50ad-TA exhibit multiple mutations in WNT and RAS/PI3K pathways, and hypermutation-associated genes, such as mismatch-repair gene MLH1 and PMS2 [12, 19], and DNA polymerase epsilon POLE, which has frequent mutations in hypermutated CRC tumors [12]. Surprisingly, two high mutation adenoma samples (MDA50ad-N/T) and MDA51ad-N/T) show frequent mutations in TGF-β and CEA-related genes, suggesting that these two adenomas could be on the brink of overt CRC (Fig 1C). The mutations in CEA-related genes in MDA50ad-N/T are in the CEA (gene *CEACAM5*) at position 84 (an Alanine-to-Threonine mutation transversion) and in CEACAM6 at residue 307 (a Glycine-to-Cysteine transversion). The adenoma genomic profiles revealed distinct mutational signatures regardless of adenoma histopathology, location, or adenoma size (Fig 1C and S1 Dataset).

Next, we looked for variants in all genes in the sequencing data. Among all the adenoma samples we observed that six genes had a high mutation frequency. These genes include APLF (Aprataxin and PNKP like Factor), KLK11 (Kallikrein-Related Peptidase 11), LAMA5 (Laminin, Alpha 5), MUC2 (Mucin 2), SIX5 (SIX Homeobox 5), and WDR90 (WD Repeat Domain 90) which are present in 36% of samples (Fig 1D, upper panels). Surprisingly, these genes are not frequently mutated in the hypermutated (0–8.6%) and non-hypermutated (0–1.1%) TCGA CRC carcinoma cohort [12] (Fig 1D, lower panel). Compared with proximal adenomas, the mutation frequencies of these genes are more significant in the distal adenomas, suggesting that they may play a barrier function in distal adenomas. Comparison of the mutational spectrums in our cohort to a set of 20 commonly mutated genes characterized in the TCGA CRC carcinoma cohort indicates that APC mutations are found in both tubular adenoma (MDA49ad-TA) and sessile serrated adenoma (MDA51ad-SSA) adenomas (Fig 1C). A KRAS mutation is observed in tubular adenoma (MDA1ad-TA) (S3A Fig). Several well-established cancer mutated genes such as TP53, PIK3CA, SMAD4, BRAF, and NRAS are not observed in our cohort. This may be either due to later stage mutations or the small sample size (S3A Fig and S1 Dataset) [12].

**Fig 2:**
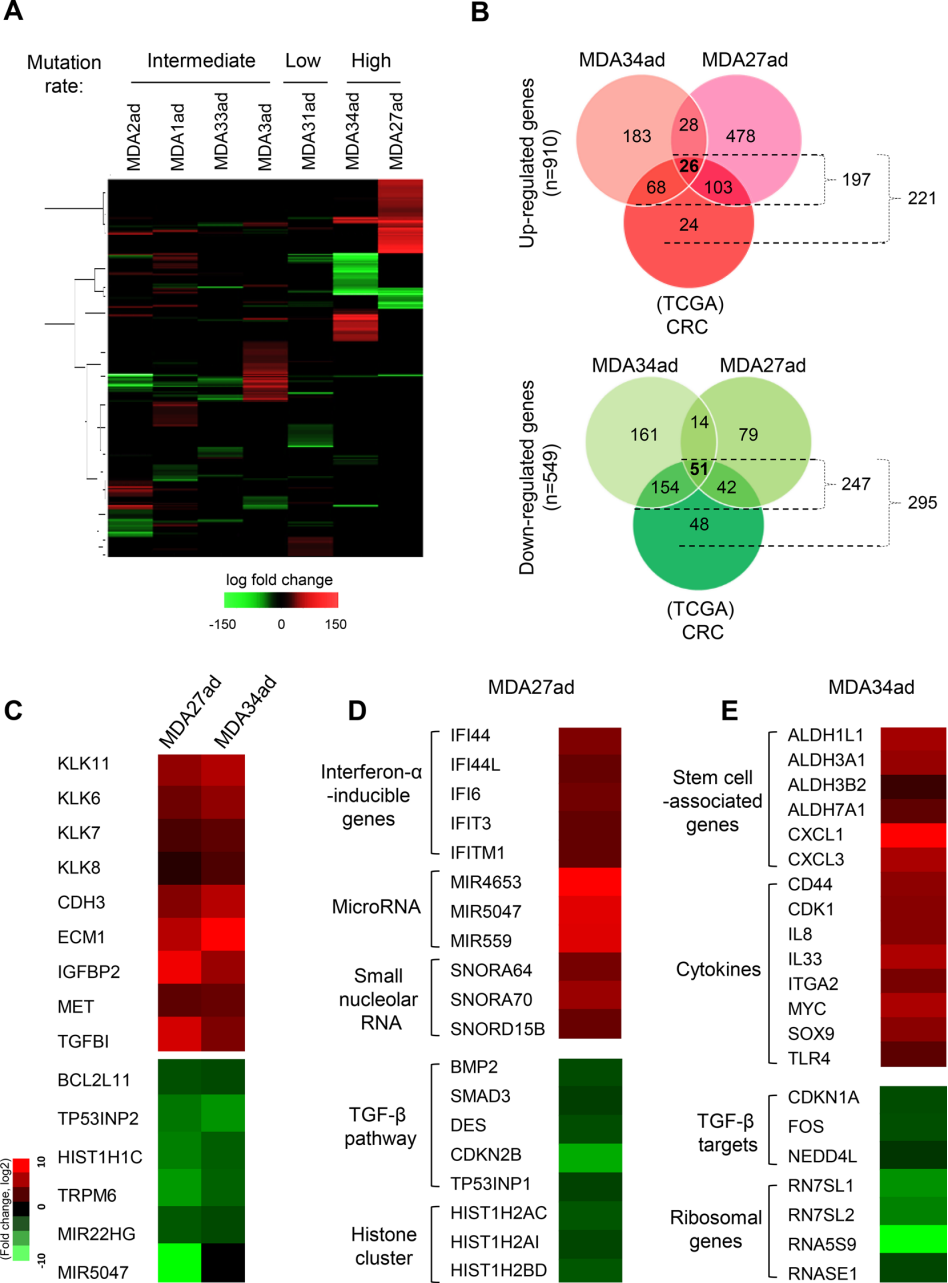
Unsupervised hierarchical clustered transcriptomic analysis reveals distinct gene expression signatures. (A) The clustering heat map reveals a unique cluster of gene signatures in two of the highly mutated adenoma samples, MDA34ad-TVA and MDA27ad-TA. The heat map was obtained using the 7 adenoma samples with the Ward hierarchical clustering algorithm and Euclidean distance metrics. The samples are classified as intermediate, low, and high based on the mutation rates observed. A unique cluster of genes was identified in two of the highly mutated adenoma samples, MDA34ad-TVA and MDA27ad-TA. (B) Venn diagram representing the CRC TCGA data set and the 2 highly mutated adenoma samples which show the unique cluster of genes. The red Venn diagram represents the genes (n = 910) that are up-regulated among the 2 highly mutated adenoma samples and the TCGA data set while the green indicates the genes (n = 549) that are down-regulated as observed among the 2 highly mutated adenoma samples and the TCGA data set (cut-off, log2fold change ± 1.5). (C) The commonly altered genes which are observed in two adenoma samples, MDA27ad-TA and MDA34ad-TVA. The representative gene expressions are shown. (D) Representative altered gene expressions are shown in adenoma sample MDA27ad-TA. (E) Representative altered gene expressions are shown in adenoma sample MDA34ad-TVA.

To identify gene expression profiles in the adenomas, unsupervised hierarchical clustering was carried out for the 7 adenoma samples (cut off, p<0.05) (Fig 2A). The clustering heat map reveals a unique cluster of gene signatures in two of the highly mutated adenoma samples, MDA34ad-TVA and MDA27ad-TA. Together, the samples had a total of 910 up-regulated genes and 549 down-regulated genes (cut off, log2 fold change ± 1.5) (Fig 2B). To validate the identified gene expression in CRC, a transcriptome sequencing data set for CRC of 262 samples was downloaded from the CRC TCGA cohort and 224 of these samples were analyzed (S3B Fig). We found that the majority of genes with altered expression profiles in MDA34ad-TVA and MDA27ad-TA adenomas correlate with the profiles in CRC. Of the genes with increased expression profiles from MDA34ad-TVA and MDA27ad-TA, 98% (197/221) were also up-regulated in TCGA CRC; of those genes with decreased expression profiles, 83% (247/295) were down-regulated in CRC (cut-off, log2fold change ± 1.5) (Fig 2B).

Among the unique gene expression signatures identified in both the two adenoma samples and the CRC TCGA cohort, we observe 26 commonly activated genes and 51 commonly inactivated genes (Fig 2B). The commonly increased genes in these two adenoma samples include KLK11 (Fig 2C), which is also mutated in 4 of 11 adenoma samples. The genes CDH3, ECM1, IGFBP2, and MET, which are associated with cell adhesion, the extracellular matrix, and cancer cell invasion/metastasis, are also observed frequently increased in these two adenoma samples (Fig 2C). The commonly repressed genes in these two adenoma samples include BCL2L11, TP53INP2, HIST1H1C, TRPM6, MIR22HG, and MIR5047 (Fig 2C). In MDA27ad-TA, there is decreased expression of the TGF-β pathway members, including BMP2 and SMAD3, as well as TGF-β/SMAD3’s targets such as DES and CDKN2B (Fig 2D). In MDA34ad-TVA, there is a similar decrease in the expression of TGF-β/SMAD3’s targets, such as CDKN1A, FOS, and NEDD4L (Fig 2E). Surprisingly, we also find that some stem cell-associated genes such as ALDH1L1 and CXCL1/3 are increased in the MDA34ad-TVA adenoma sample, suggesting a stem cell phenotype (Fig 2E) [8, 20]. Unsupervised hierarchical clustering of CRC TCGA data sets was also performed. The clustering heat map of CRC carcinoma samples shows that 26 commonly activated genes observed in adenomas are markedly increased (in Cluster 1 and 2), whereas 3 commonly inactivated genes are dramatically decreased (in Cluster 3) (S3B Fig).

**Fig 3:**
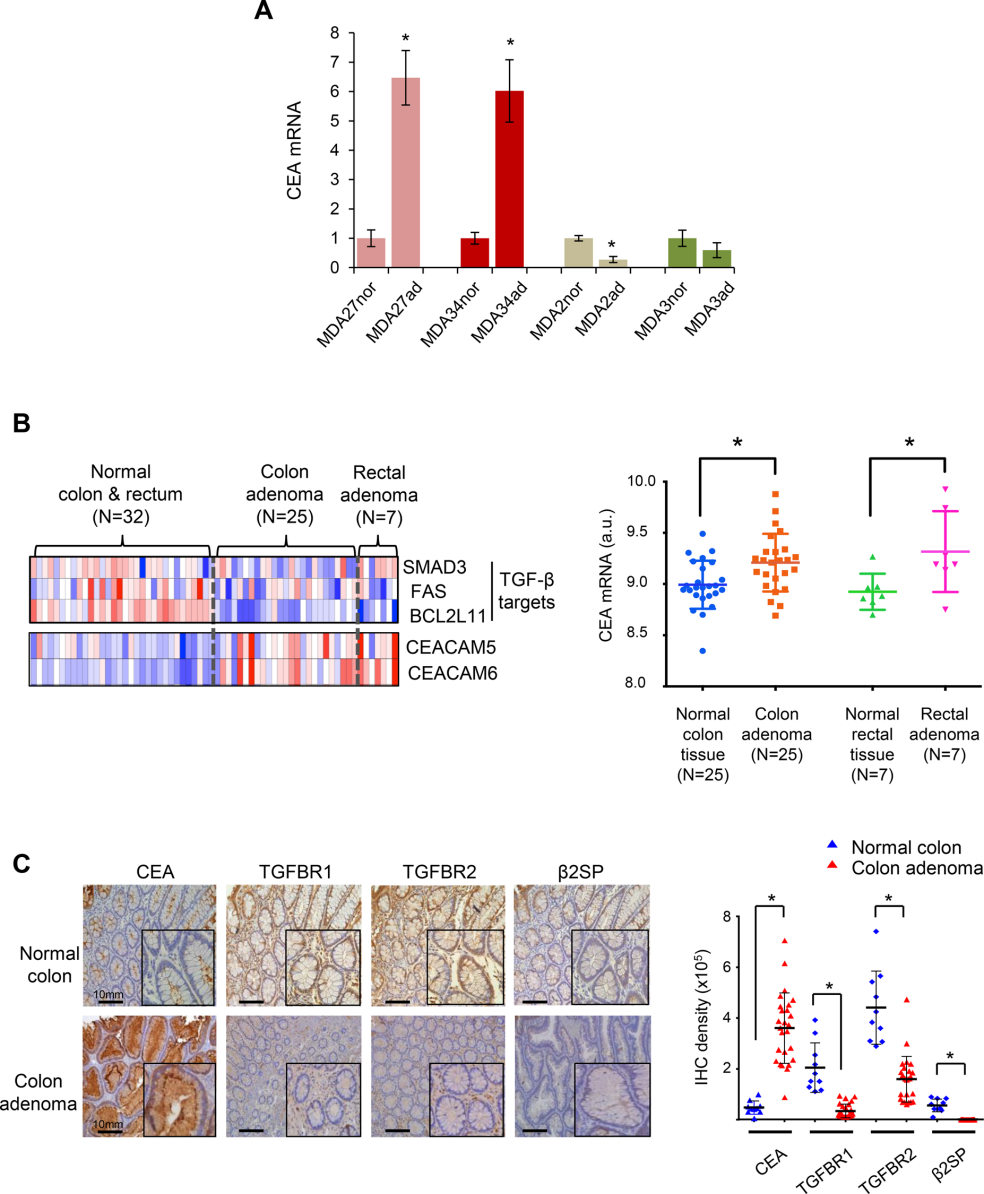
Enhanced expression of CEA correlates with loss of TGF-β signaling in early colon adenomas. (A) Marked increases in CEA mRNA expression levels in the highly mutated adenoma samples, MDA27ad-TA and MDA34ad-TVA. CEA mRNA expression levels were detected in 4 adenomas (MDA27ad-TA, MDA34ad-TVA, MDA2ad-TVA, and MDA3ad-SSA) and 4 matched normal mucosa samples (MDA27nor, MDA34nor, MDA2nor, and MDA3nor) by Q-RT-PCR. Results are the average of three independent experiments and are presented as mean ± SD. *p < 0.01, versus normal tissues, Student’s t-test. (B) Analysis of an Oncomine™ dataset demonstrates high CEA mRNA levels in colon and rectal adenomas compared to that in corresponding normal colorectal tissue. CEA and CEACAM6 mRNA expression is inversely associated with TGF-β target gene levels in normal and adenoma colorectal tissues. Transcriptomic profiles of 32 colorectal adenoma tumors and their 32 corresponding normal colorectal tissue were downloaded from Gene Expression Omnibus database (data set GSE8671). These gene expression profiles were then analyzed using Oncomine analysis tools (www.oncomine.org). Data are displayed as a heat map using an Oncomine™ graphic platform and as a dot plot using a GraphPad Prism v5.0 program. *: *p* < 0.05, Student’s t-test. (C) Adenoma samples analysis of CEA, TGFBR1, TGFBR2, and β2SP reveals a negative correlation between CEA and TGF-β genes in colon adenomas. Sections from human clinical samples of normal (n = 10) and adenoma colon tissue (n = 26) were prepared and processed for immunohistochemical analysis, with further confirmation using 40 adenomas. Magnification × 20; insets magnification × 60. Scale bars, 10 mm. Quantification of the immunohistochemical staining is shown. Mean ± SD is shown *: *p* < 0.01, Student’s t-test.

CEA (gene *CEACAM5*), a 180kDa GPI-linked membrane glycoprotein, belongs to a subgroup of the immunoglobulin supergene family CEACAMs [21, 22]. Studies have shown that CEA and CEACAM6 levels are elevated in colon cancer patients [23–25]. We observed markedly increased CEA (CEACAM5) levels in the highly mutated adenoma samples MDA34ad-TVA and MDA27ad-TA, compared with matched normal mucosa samples (Fig 3A). Additionally, analysis of an Oncomine^TM^ dataset demonstrates high CEA mRNA levels in colon and rectal adenomas (Fig 3B). Despite extensive investigation and identification of other pro-tumorigenic CEACAMs, the functional role of the CEACAMs in CRC progression is poorly understood. We have previously shown that CEA associates with TGF-β receptor 1 (TGFBR1) and inhibits TGF-β signaling which increases CRC liver metastasis [24]. Because we also observed frequent mutations in TGF-β signaling pathways in adenomas, we sought to determine a potential association between the expression levels of CEA and members of the TGF-β signaling pathway including TGFBR1, TGFBR2 and Smads adaptor β2SP in 26 early adenomas and 10 normal colon tissues (Fig 3C). We observed a marked increase in CEA expression (nearly 5-fold) in 23% of adenoma samples with a concomitant loss of TGF-β signaling confirmed by a further analysis of 40 samples.

**Fig 4:**
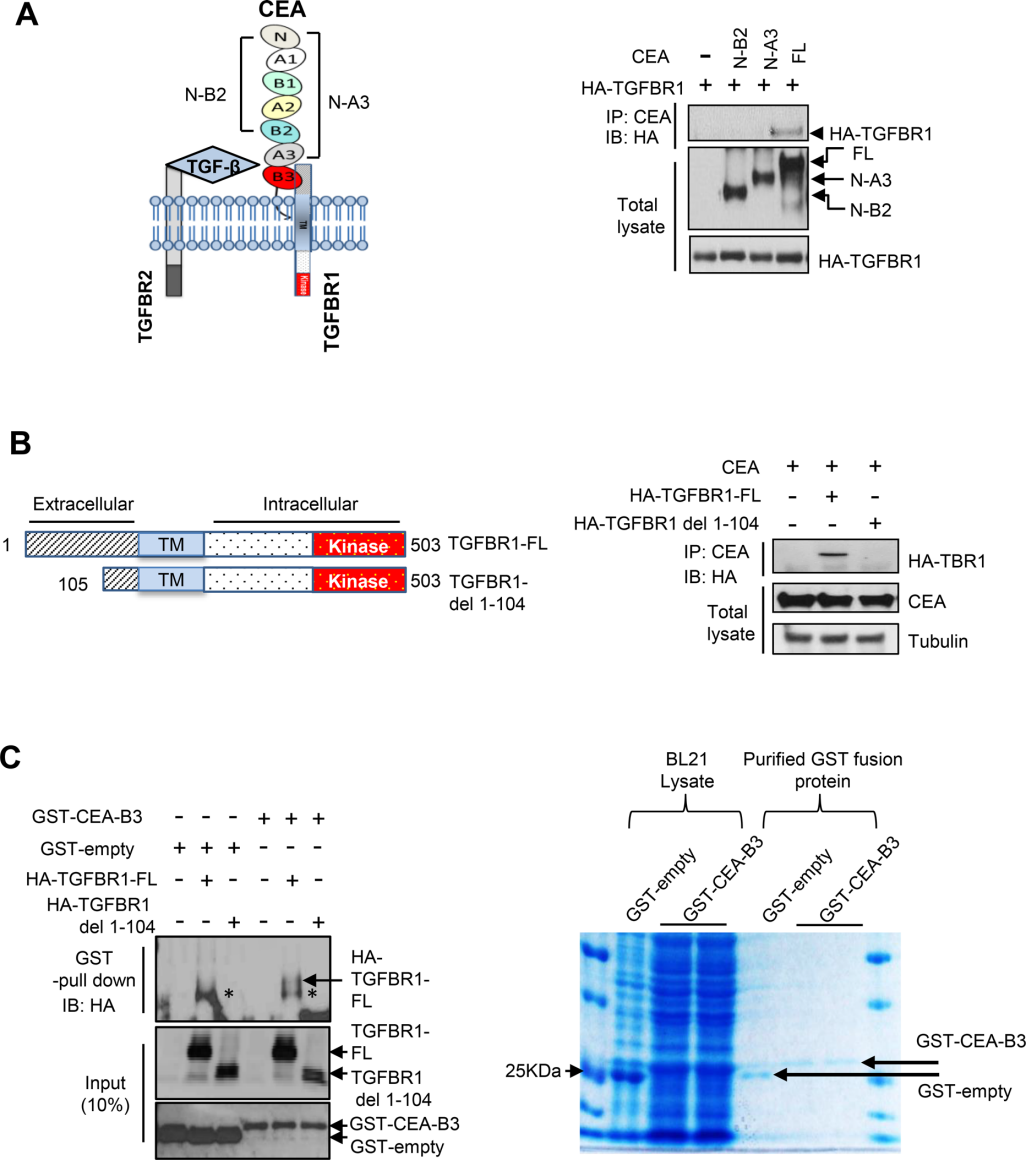
CEA interacts with the TGF-β pathway. (A) The CEA B3 domain is required for the interaction of CEA with TGFBR1. HCT116 cells were co-transfected with the generated mutants. Cell lysates were immunoprecipitated with the CEA antibody and were immunoblotted with the indicated antibodies. (B) CEA interacts with the extracellular domain of TGFBR1 (amino acid 1–104). The generated TGFBR1 mutant plasmids were co-transfected into the cell lines and immunoprecipitation was carried out with CEA antibodies, followed by immunoblotting. (C) The CEA B3 domain interacts directly with TGFBR1. The CEA B3 domain GST fusion protein was produced bacterially. HA-TGFBR1-FL or HA-TGFBR1-1-104 deletion plasmids were transfected in 293T cells. Purified TGFBR1-FL (full length) or HA-TGFBR1- del-1-104 was incubated with GST-empty or GST-CEA-B3 proteins. The binding of TGFBR1 to CEA-B3 domain was detected by immunoblotting using anti-HA antibody. * designates non-specific bands. Purified GST fashion proteins were detected by Coomassie blue staining. Glutathione Resin GST Fusion Protein Purification Kit (GenScript, Cat. L00206) was used for purification of GST-CEA-B3 domain fusion protein.

We next explored how increased CEA levels could disrupt the TGF-β pathway by examining the physical interactions between CEA and TGFBR1. For this, we generated a series of CEA and TGFBR1 deletion mutants (Fig 4A, left panel). Immunoprecipitation assays demonstrate that the B3 domain of CEA directly interacts with TGFBR1 (Fig 4A, right panel), and that the N-terminus domain of TGFBR1 (amino acids 1–104 of the extracellular domain) is required for interacting with CEA (Fig 4B). In vitro binding assays reveal that the CEA B3 domain interacts directly with TGFBR1 (Fig 4C).

**Fig 5:**
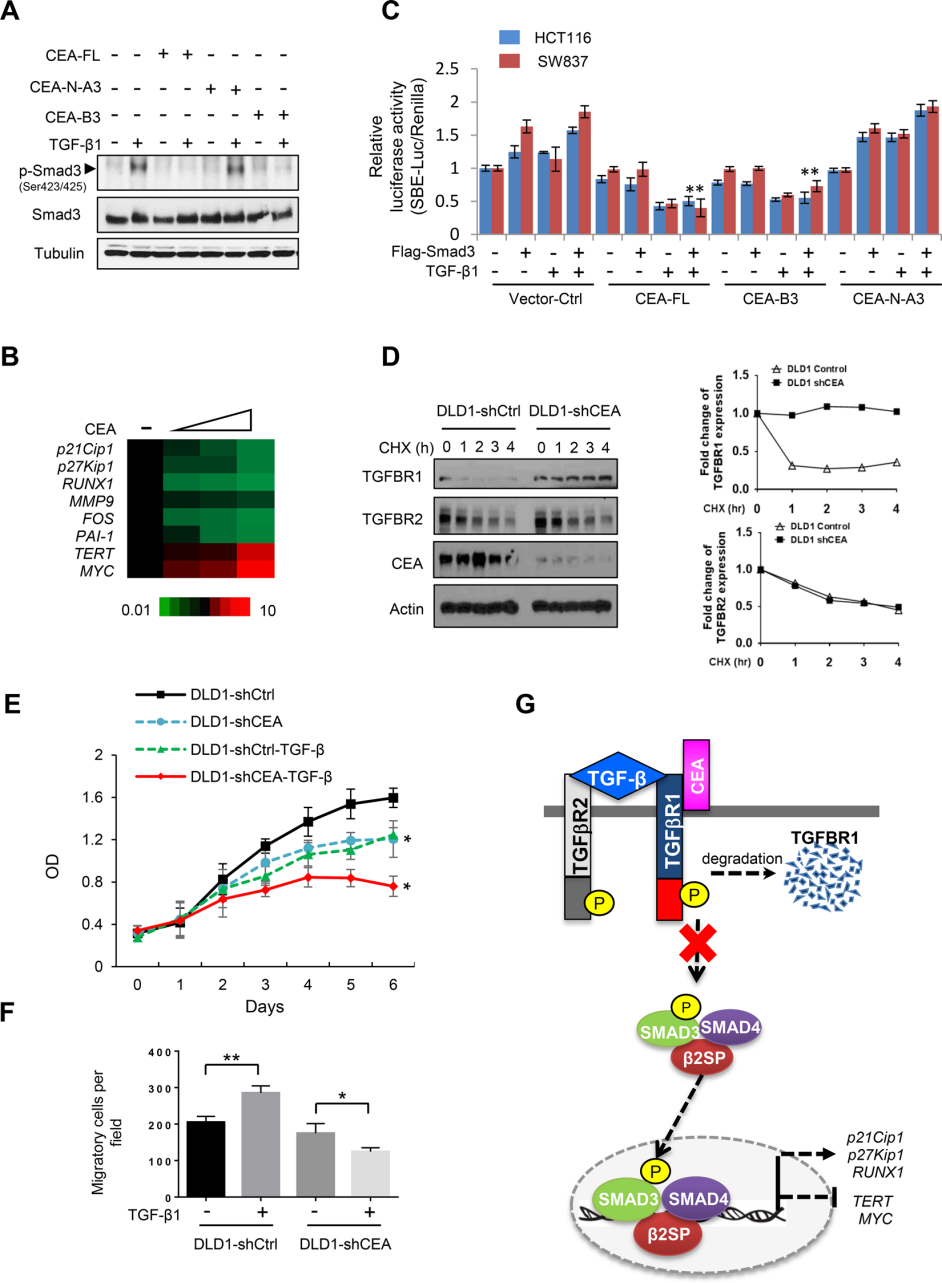
CEA interacts with and disrupts the TGF-β pathway. (A) Overexpression of CEA B3 domain prevents TGF-β-induced Smad3 phosphorylation. HCT116 cells were co-transfected with CEA wild type (FL) or various CEA deletion mutants. The cells were stimulated with TGF-β1 for 2 hours. The cell lysates were immunoblotted with the indicated antibodies. (B) CEA transcriptionally regulates TGF-β pathways. HCT116 cells were transfected with CEA. The mRNA levels of TGF-β targets were detected by Q-RT-PCR. The result shown is representative of three independent experiments. (C) Overexpression of CEA B3 domain inhibits Smad3 transcriptional activity. HCT116 or SW837 cells were co-transfected with Smad3 luciferase reporter plasmids, CEA (FL, N-A3, and B3), and Renilla luciferase reporter plasmids. The cells were stimulated with TGF-β1 for 2 hours. Cell lysates were collected and analyzed according to the manufacturer’s protocol. Results are the average of three independent experiments and are presented as mean ± SD. *p < 0.01, compared with Flag-Smad3/TGF-β1 treatment in Vector-Ctrl cells. Student’s t-test. NS: not significant. (D) CEA knockdown increases TGFBR1 protein stability. DLD1 cells were knocked down with control or shCEA. Cells were treated with 100ug/ml cycloheximide (CHX) for the indicated times. The density of TGFBR1 or TGFBR2 and the integrated optical density were measured. The turnover of TGFBR1 or TGFBR2 is indicated graphically. (E) Knock down CEA and/or TGF-β treatment suppresses CRC cell growth. Cell proliferation was assessed by colorimetric MTS assays. Results are the average of three independent experiments and are presented as mean ± SD. *p < 0.01, Student’s t-test. (F) TGF-β significantly suppresses cell migration in DLD1 CEA knockdown cells, while increasing cell migration in control DLD1-shCtrl cells. Transwell migration assays of DLD1-shCtrl or DLD1-shCEA cells were performed. Cells were treated with TGF-β1 (200pM) for 24 hours. Results are the average of three independent experiments and are presented as mean ± SD. *p < 0.05, **p < 0.01, Student’s t-test. (G) Proposed model of the role of CEA in the regulation of TGF-β pathway.

Further analysis demonstrates that the B3 domain of CEA inhibits TGF-β-stimulated phosphorylation of Smad3 (Fig 5A). Over expression of CEA in HCT116 cells significantly alters TGF-β/Smad3 transcriptional targets as evidenced from the Q-RT-PCR assays (Fig 5B). The luciferase reporter assays demonstrate that the B3 domain of CEA inhibits TGF-β-induced Smad3 transcriptional activates in both HCT116 and SW837 cells (Fig 5C). Knockdown of CEA in colon cancer DLD1 cells increased TGFBR1 protein stability, suggesting that CEA may regulate TGFBR1 levels post-transcriptionally (Fig 5D). Also, we examined the effect of CEA knockdown on the proliferation and migration in DLD1-shCtrl and DLD1-shCEA CEA knockdown cells with and without TGF-β treatment. Our data indicate that knockdown CEA in CRC cells significantly suppresses cell growth. TGF-β treatment of CEA knockdown cells has more marked inhibitory effect on cell proliferation (Fig 5E). Strikingly, TGF-β significantly suppresses cell migration in DLD1 CEA knockdown cells, while increasing cell migration in control DLD1-shCtrl cells (Fig 5F). The data suggest that TGF-β plays a tumor suppressor function in the context of loss of function of CEA in these CRC cells. Taken together, these results support that CEA disrupts the TGF-β pathway by interfering with TGFBR1 stability, which in turn blocks the Smad3 phosphorylation cascade and its downstream transcriptional targets (Fig 5G).

## Discussion

Although there are 3 effective clinical chemo-preventive agents (i.e., Vitamin D/calcium, estrogen-containing hormone replacement therapies, aspirin/NSAIDs/COXIBs) [26], these are used less frequently than expected because of their limited efficacy and/or high toxicity. However, these agents provide proof of principle for chemoprevention in CRC and suggest important concepts on which to build future developmental studies. The current cancer risk prediction models mostly rely on environmental risk factors and so have modest discriminatory accuracy. Our approach towards improved risk prediction incorporates genetic information and biomarker-driven models used in conjunction with animal models. For other cancers, intermediate phenotypic biomarkers have yielded improved risk prediction. For instance, mutagen sensitivity in lymphocytes has been used as an indirect measure of latent genetic instability. Yet, there still have been no concerted efforts thus far to identify intermediate phenotypic biomarkers in CRC risk prediction. The largest gap of knowledge is the lack of integrative risk models for any stage of the CRC continuum. We begin addressing these issues through our analysis of normal/neoplastic tissues from individuals with pre-invasive colonic neoplasms that are suspected of developing a premalignant genome analysis. Our studies further support an important functional role for CEA and TGF-β signaling in early benign adenomas (polyps) that give rise to CRC.

Surveillance colonoscopy has been advised every 3–5 years for individuals with a history of adenomas; individuals with an adenoma-free history are recommended to do so every 10 years. This creates a large burden on surveillance colonoscopy, which accounts for 25% of the total colonoscopies performed annually in the United States [5]. Considering this, there is an urgent need to develop new techniques which will facilitate early detection of the disease. Conventionally, high-risk adenomas are defined clinically as those that are larger than 1 cm. These adenomas are considered to give rise to overt carcinomas and are an important criterion in colonoscopy and screening studies [3, 4, 6, 7]. We attempted to identify high-risk genetic profiles of adenomas which could develop to rapid-onset CRC. We describe the genetic analysis of four sets of colonic adenoma tissue samples paired with their normal counterparts using whole-genome sequencing analysis. We extend this to transcriptomic analysis of seven further pairs of adenomas. We propose that our observations of several types of precancerous mutations and transcriptomic profiles in 4 out of the 11 adenomas may be used clinically to predict higher risk of transformation to CRC. In one of the two samples, surprisingly high mutational profiles of multiple genes, including CEA and CEACAM6, is demonstrated. Transcriptomic analyses and immunohistochemical analysis of adenoma tissue samples further demonstrate enhanced CEA expression and concomitant loss of TGF-β signaling components in 23% adenoma samples.

TGF-β is a key regulator of multiple biological processes, including cell proliferation, differentiation, migration, apoptosis [27, 28], and tumor suppression in early stages of cancer. Yet, in later stages it is pro-metastatic [29]. Current data support TGF-β signaling as a suppressor of early colorectal cancers [30, 31]. In later stages of the disease, metastatic CRCs escape the tumor-suppressor effects of TGF-β signaling by becoming both resistant to TGF-β-induced growth inhibition and harnessing the pro-tumorigenic properties of TGF-β [24]. Inactivating mutations in TGFBR2 occur in 87% of human colorectal carcinomas with microsatellite instability [32].

Recent studies have demonstrated that CEACAM functions are pleiotropic, including intercellular adhesion [22, 33], modulation of cell homeostasis [34], T-cell proliferation [35, 36], neovascularization. Also, CEACAM functions as a receptor for host-specific viruses and bacteria that harness the CEACAMs for successful host colonization [37]. CEA and CEACAM6 are considered to be pro-tumorigenic in colon cancer. CEA has been utilized as a marker for recurrent disease in CRC, and also in thyroid and pancreatic cancer [38, 39]. While CEA vaccines have been utilized in advanced CRC, they have yet to be studied in the prevention arena; thus, our study warrants a large cohort analysis of vaccination and prevention measures in high-risk populations of patients with colon adenomas that have aberrant TGF-β signaling concurrent with overexpression of CEA. Collectively, our analysis may provide an important approach to prevent CRC and identify high-risk patients with surprisingly high mutational profiles or who expresses high CEA earlier than previously thought. Further work may lead to a new paradigm in the cost-effective prevention of CRC.

## Materials and Methods

### Patients and samples

We performed whole-genome sequencing on 4 pairs of adenoma samples and whole-transcriptome RNA sequencing on 7 matched colon adenoma samples and normal mucosa obtained from the University of Texas, MD Anderson Cancer Center (GSE72820).

We analyzed the transcriptome (n = 262) and screened for mutations (n = 224) in CRC patients from The Cancer Genome Atlas (TCGA) downloaded on November 2014. In addition, transcriptomic profiles of 32 colorectal adenomas tumors and their 32 corresponding normal colorectal tissues were downloaded from Gene Expression Omnibus database (GSE8671). These gene expression profiles were then analyzed using Oncomine™ analysis tools (www.oncomine.org). Data were displayed as a heat map using an Oncomine™ graphic platform and as a dot plot using a GraphPad Prism v5.0 program. The study was approved by the Office of Human Subjects Research of the National Institutes of Health and MD Anderson Cancer Center, and all samples were de-identified.

### Cell culture, transfection, and shRNA-mediated silencing

Human colon epithelial cell line DLD-1 (ATCC, CCL-221), HCT 116 (ATCC® CCL-247™) and SW837 (ATCC® CCL-235™) were cultured in DME/F12 medium, and (Sigma Aldrich, D5671) supplemented with 10% fetal bovine serum (Sigma Aldrich, F2442). Transfection of CEA plasmid constructs was carried out using Lipofectamine 2000 (Invitrogen). Lentiviral particles containing CEA (sc-36551) or control shRNA lentiviral particles (sc-108080) were purchased from Santa Cruz Biotechnology and were used to infect DLD1 cells.

### Cell growth and viability assays

Cell proliferation was assessed by a colorimetric MTS assay according to the manufacturer's instructions (Cell Titer 96 Aqueous One Solution Cell Proliferation Assay; Promega, Madison, WI, USA, Ca# G3580). A total of 5 × 10^3^ cells were seed in 96 well plates at day 0. Beginning the next day, cells were treated with TGF-β1 (200pM) each day (day 1 to day 6). For measuring cell numbers, the cells were incubated for 3 h with MTS solution. Subsequently, the absorbance was measured at 490 nm on an ELISA reader (BioRad Model 550).

### Immunohistochemical analysis

Sections from human clinical samples of normal (n = 10) and adenoma colon tissue (n = 26) were prepared and processed for immunohistochemical analysis using antibodies against CEA, TGFBR1, TGFBR2, and β2SP. Staining intensities were measured between the normal and adenoma samples in at least five different fields. Protein expression was objectively and quantitatively measured using an ACIS III automatic cellular imaging system (DAKO) equipped with automatic microscopy and advanced computerized image analysis.

### Construction of the plasmids and stable cell lines

cDNA sequence of TGFBR1 was amplified by gene-specific primer and inserted into pCMV-HA mammalian expression vectors (pCMV-HA) (Clonetech). To construct pCMV-HA-TGFBR1 (del-1-104), N-A3, N-B2 and B3 domain of CEA, cDNA sequence of each gene was amplified by site-specific polymerase chain reaction, and then subsequently subcloned into the same mammalian expression vector. To generate a stable shCEA cell line, HEK293T cells were transfected with the indicated lentiviral plasmid DNAs to make lentiviral particles through the viral packaging process. Virus-containing supernatants were collected and filtered, then DLD1 cells were infected with lentiviral particles, either shLuciferase or shCEA with 8 μg/ml of polybrene. After infection, cells were selected with 2–4 μg/mL of puromycin for 2 weeks.

### Immunoprecipitation

To analyze the interaction of CEA with TGFBR1, HCT116 cells were co-transfected with FL, N-B2 and N-A3 CEA with either HA-TGFBR1-FL or HA-TGFBR1-del 1–104 plasmid DNA. Cells were incubated for 48hrs, and were lysed with lysis buffer [20mM Tris (Fisher), 100mM NaCl (Fisher), 0.5% Nonidet P-40 (USB Corp.), 0.5% Triton X-100 (Sigma), 1mM EDTA (Fisher)]. Fresh protease/phosphatase inhibitors [5 mM NaV, 1 mM NaF, 1 μM DTT, 0.1 mg/mL Pepstatin A, 1 mM PMSF, and 1,000× Complete Mixture Protease Inhibitor (Roche)] were added into the lysis buffer. For IP, lysed cells were incubated with CEA antibody (Thermo MS-613-P0) for 2 hours at 4°C and then incubated with protein G plus agarose beads (Amersham) for 2 hours at 4°C. Beads were washed with TBST and were subjected to immunoblotting.

### GST pull-down and in vitro binding assays

BL21 cells were transformed with pGEX-2TK or pGEX-2TK-CEA-B3 and incubated with 0.2 mM IPTG for 4 hours. The GST fusion proteins were purified from bacterial lysates with GSH-Sepharose 4B beads according to the manufacturer’s instruction (GenScript, Cat. L00206). 293T cells were transfected with HA-TGFBR1-FL or HA-TGFBR1-del-1-140 and HA fusion proteins were immunoprecipitated and purified from cell lysates using HA-tagged protein purification kit (MBL, 3320A). For the GST pull-down assay, cell lysates were prepared by sonication and spun at 15,000 rpm for 15 min. The supernatants were incubated with GST sepharose beads for 3 h at 4°C. For the in vitro binding assay, purified HA-TGFBR1-FL or HA-TGFBR1-del-1-140 was incubated with a GST or GST- CEA-B3 fusion protein that conjugated to Sepharose beads in 500 μl of reaction buffer (50 mM Tris-HCl, pH 7.5, 150 mM NaCl, 5 mM NaF, 1% NP40, and protease inhibitor mixture) for 12 h at 4°C. After centrifugation, the proteins bound to Sepharose beads were washed with cold PBS, mixed with SDS sample buffer. The binding of TGFBR1 to CEA-B3 domain was detected using anti-HA antibody.

### In vitro migration and invasion assay

For transwell migration assays, 1 × 10^5^ cells were plated in the top chamber with the non-coated membrane (24-well insert; Cat. 3422, Corning). The cells were plated in medium without serum or growth factors, and medium supplemented with serum was used as a chemoattractant in the lower chamber. Cells were incubated for 24 hours with or without TGF-β1 (200pM) and cells that did not migrate through the pores were removed by a cotton swab. Cells on the lower surface of the membrane were stained with the Diff-Quick Staining Set (Dade) and counted.

### Western blotting and antibodies

Cells were lysed with lysis buffer and were standardized using a BCA protein assay (Pierce™ BCA Protein Assay Kit 23225). Equal amounts of proteins were fractionated on SDS–PAGE and blotted to nitrocellulose membrane. Membranes were incubated with CEA (Thermo MS-613-P0), TGFBR1 (Santa Cruz Sc-398), TGFBR2 (ab17650), Phospho-Smad3 (Ser423/425) (Cell Signaling #9520), Smad3 (Cell Signaling #9523), and tubulin (Cell Signaling #2144) antibodies. Secondary antibodies conjugated with horseradish peroxidase (Chemicon) and Enhanced chemiluminescence (ECL) kit (Perkin-Elmer Life Sciences) were used to develop the immunoblots.

### Luciferase assays

To analyze Smad3 transcriptional activity, HCT116 cells or SW378 cells were seeded at a density of 1x10^4^ cell/well in 24-well culture dishes. Cells were co-transfected with Smad3 luciferase reporter plasmid, CEA (FL, N-A3 and B3) and Renilla luciferase reporter plasmids. After transfection, cells were incubated in a serum-free media for 24h and were treated with either TGF-β1 or vehicle and then incubated for a further 2 hours. At harvest, cells were collected using passive lysis buffer (Promega) and analyzed according to the manufacturer’s protocol for the Dual Luciferase Reporter Assay kit (Promega).

### Statistical analyses

Pairwise comparisons were performed using Student’s t-test * P < 0.01. Graphs represent mean ± standard deviation unless otherwise noted.

## Supporting Information

S1 Dataset Somatic mutations in adenoma samples. (XLSX)

S1 Fig Histologic specimen of human colorectal adenoma tissue stained with hematoxylin and eosin. Eleven pairs of matched colon adenoma samples and normal mucosa were obtained from the University of Texas, MD Anderson Cancer Center. Four pairs were for whole-genome sequencing and a further 7 pairs were for whole-transcriptome RNA sequencing analyses.
(TIF)

S2 Fig Frequent mutations are observed in colon adenomas by WTS. (A) Mutation frequency detected by whole-transcriptome (WTS) sequencing of colon adenoma tissues. The dot represents the number of mutations per Mb in one adenoma sample. The red and orange dots represent the median mutation per Mb as observed in the colorectal cancer samples from the TCGA database. (B) Transitional single nucleotide substitutions of C:G > T:A predominate in the adenoma mutational spectrum.
(TIF)

S3 Fig Gene profiles of the adenomas. (A) The top 20 common gene mutations observed in the CRC TCGA data set were examined in 11 colon adenoma tissues (left panel). The overall percentage of mutations observed in the CRC TCGA data set is presented with divided percentages calculated for the proximal and distal (right panel). TA: Tubular adenoma; TVA: Tubulovillous adenoma; SSA: Sessile serrated adenoma. Location d: distal; p: proximal. (B) A heat map of the CRC TCGA data set with 26 commonly activated and 3 most commonly inactivated genes which were observed in both the 2 adenoma samples MDA34ad-TVA and MDA27ad-TA, and the CRC TCGA samples. Three clusters are shown, two from the up-regulated clusters and one from the down-regulated cluster. The significant genes from the clusters are represented (cut off, p<0.05).
(TIF)

S1 Methods (DOCX)

S1 Table Mutation frequency of adenoma samples. (DOCX)
