# Supplementary figures and images for: Mutational Profiles Reveal an Aberrant TGF-β-CEA Regulated Pathway in Colon Adenomas
Source: PLoS One. 2016 Apr 21;11(4):e0153933. doi: 10.1371/journal.pone.0153933 (PMC4839765; doi:10.1371/journal.pone.0153933)

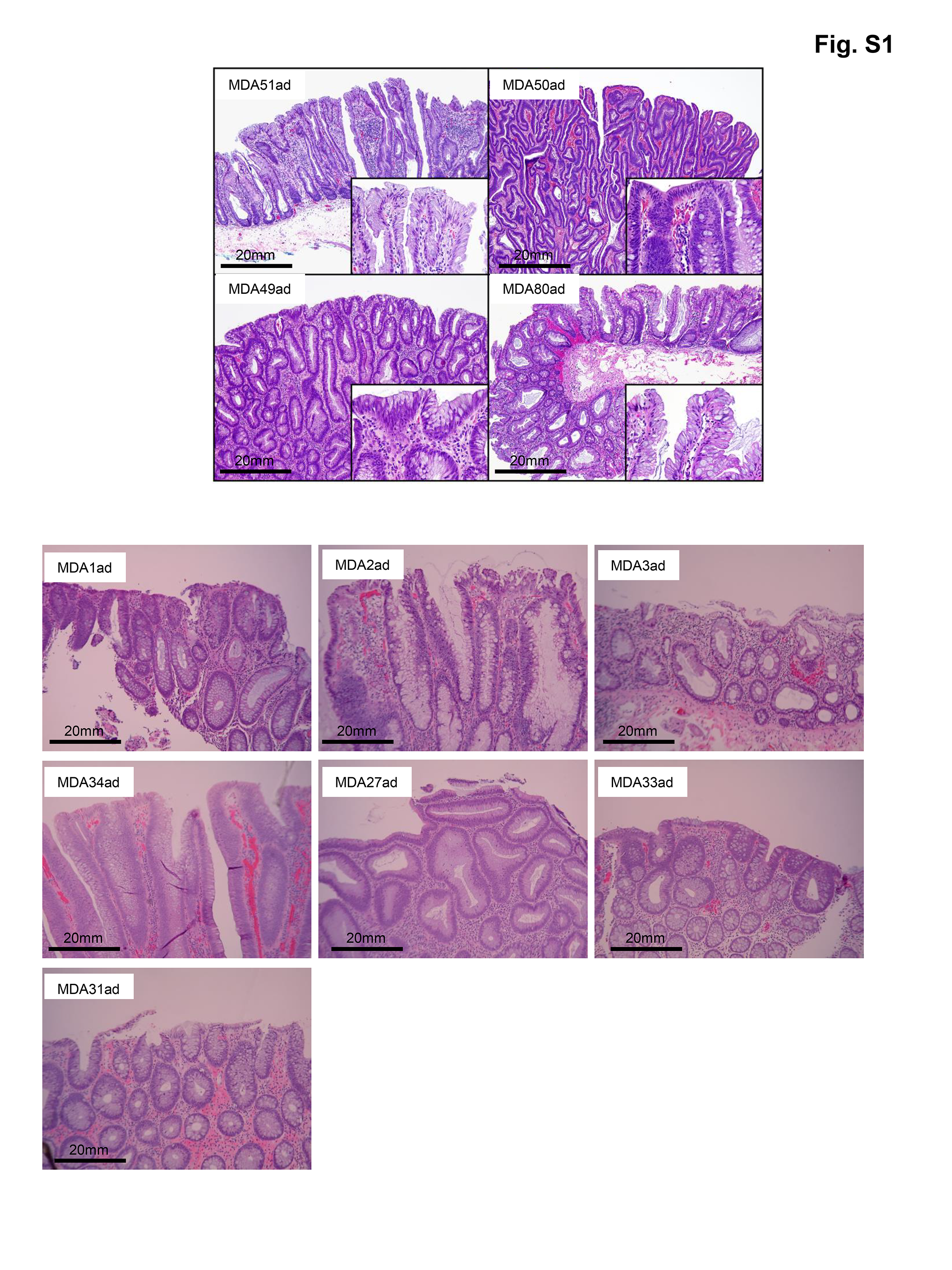

Supplement: S1 Fig — Eleven pairs of matched colon adenoma samples and normal mucosa were obtained from the University of Texas, MD Anderson Cancer Center. Four pairs were for whole-genome sequencing and a further 7 pairs were for whole-transcriptome RNA sequencing analyses. (TIF) [file pone.0153933.s002.tif]

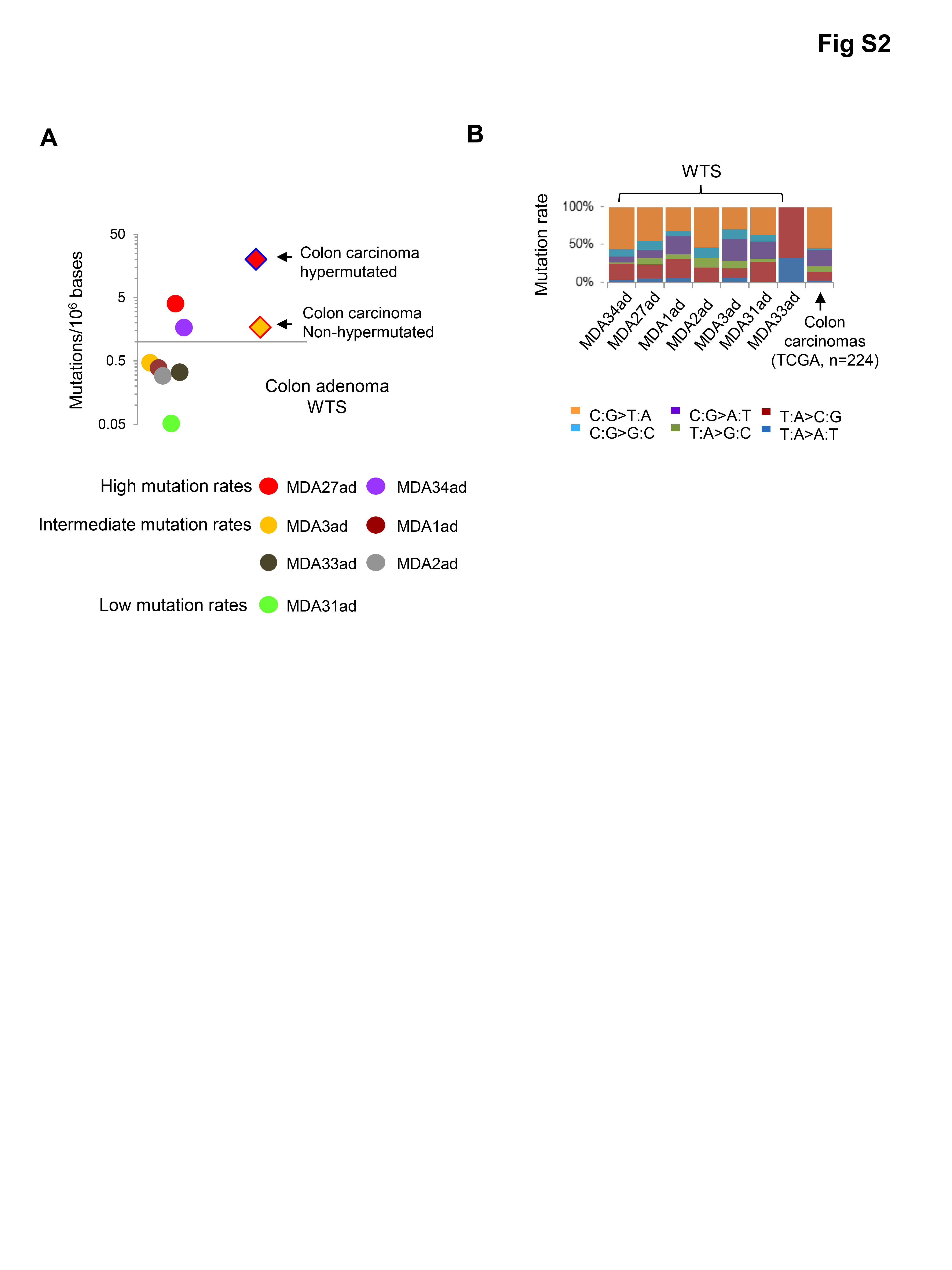

Supplement: S2 Fig — (A) Mutation frequency detected by whole-transcriptome (WTS) sequencing of colon adenoma tissues. The dot represents the number of mutations per Mb in one adenoma sample. The red and orange dots represent the median mutation per Mb as observed in the colorectal cancer samples from the TCGA database. (B) Transitional single nucleotide substitutions of C:G > T:A predominate in the adenoma mutational spectrum. (TIF) [file pone.0153933.s003.tif]

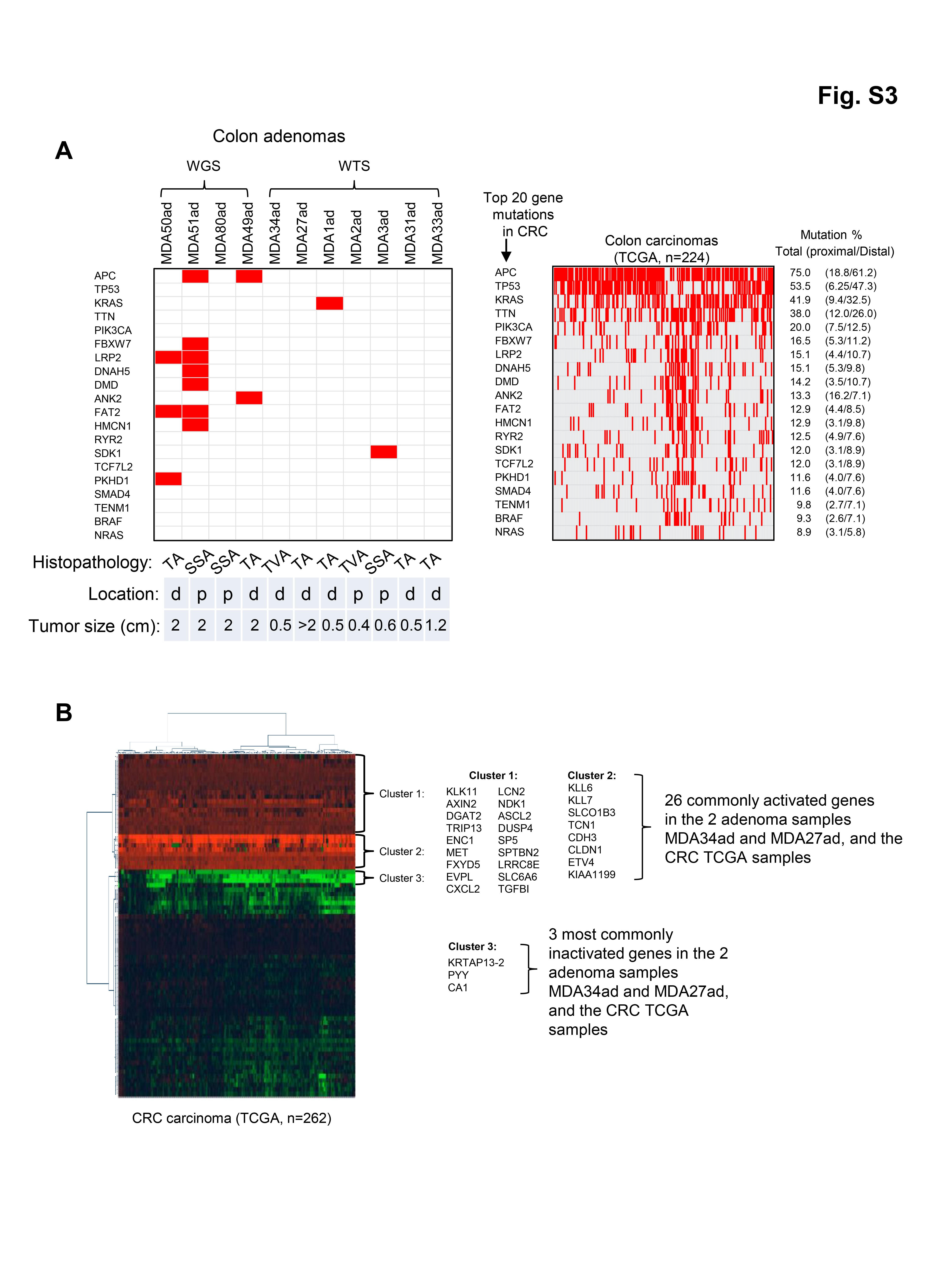

Supplement: S3 Fig — (A) The top 20 common gene mutations observed in the CRC TCGA data set were examined in 11 colon adenoma tissues (left panel). The overall percentage of mutations observed in the CRC TCGA data set is presented with divided percentages calculated for the proximal and distal (right panel). TA: Tubular adenoma; TVA: Tubulovillous adenoma; SSA: Sessile serrated adenoma. Location d: distal; p: proximal. (B) A heat map of the CRC TCGA data set with 26 commonly activated and 3 most commonly inactivated genes which were observed in both the 2 adenoma samples MDA34ad-TVA and MDA27ad-TA, and the CRC TCGA samples. Three clusters are shown, two from the up-regulated clusters and one from the down-regulated cluster. The significant genes from the clusters are represented (cut off, p<0.05). (TIF) [file pone.0153933.s004.tif]
