# Supplementary material for: Mutational Profiles Reveal an Aberrant TGF-β-CEA Regulated Pathway in Colon Adenomas
Source: PLoS One. 2016 Apr 21;11(4):e0153933. doi: 10.1371/journal.pone.0153933 (PMC4839765; doi:10.1371/journal.pone.0153933)
Supplement: S1 Methods — (DOCX) [file pone.0153933.s005.docx]

**Supplementary methods**

**Whole-genome sequencing analyses**

Whole-genome sequencing was carried out on 4 pairs of normal mucosa and colon adenoma samples. To remove duplicate reads and recalibrate base quality, sequence reads were mapped to the hg19 reference genome (http://www.ncbi.nlm.nih.gov/) using the publically available BWA tool, SAM tools, Picard (http://picard.sourceforge.net/command-line-overview.shtml) and GATK. Single nucleotide variants and index variants were identified using the Unified Genotyper caller of the GATK. Mutations were annotated with SeattleSeq Annotation (http://gvs.gs.washington.

edu/SeattleSeqAnnotation/). An SQL database was created from the annotated dataset. Somatic mutations in tumor samples were identified through comparison with the sequence of the associated normal control sample, dbSNP130, 1000Genomes, and other unrelated samples in an in-house created database. In order to be called somatic mutations, two things were required: first, at least five-fold total read coverage of the area of interest; second, mutant reads had to represent less than 2% of total reads in the normal control sample. In addition, manual examination was conducted with TViewer of SAMtools to identify high confidence mutations from the raw sequence data.

**Whole-transcriptome sequencing analyses**

Whole-transcriptome RNA sequencing for seven colon adenoma samples was performed and analysis was done at the MD Anderson Cancer Center DNA core facility.

Mapping/Alignment: The raw, paired-end reads in FASTQ format were then aligned to the human reference genome, GRCh37/hg19, using MOSAIK alignment software. MOSAIK works with paired-end reads from Illumina HiSeq 2000 and uses both a hashing scheme and the Smith-Waterman algorithm to produce gapped optimal alignments and to map exon junction-spanning reads with a local alignment option for RNA-seq. The resulting alignments were then saved as a standard bam file.

The raw counts for each gene of mRNAs from RNA-seq: We then counted the mapped reads in genomic features such as genes annotated in GENCODE15 to generate the raw counts for each gene using the HTSeq-count script distributed with the HTSeq package. We chose the “union” mode of HTSeq to mask the regions that overlapped between mRNAs and lncRNAs to overcome the issue of non-strand-specific RNA sequencing in the kit (TruSeq) used in TCGA data.

Count data normalization: Raw reads count data were normalized across samples with DESeq_1.10.1. Specifically, DESeq first estimates the effective library size, which is also called size factor, by dividing each column by the geometric means of the rows given a matrix or data frame of raw count data. Then, the median of these ratios (skipping the genes with a geometric mean of zero) are used as the size factor for that column. With the estimation of size factors, DESeq then divides each column of the count table by the size factor for that column. By doing that, the count values are brought to a common scale, making them comparable across samples. Furthermore, we transformed the count data by the Variance Stabilizing Transformation function provided in the DESeq package. With this function, the standard deviation of each gene is roughly constant regardless of the gene expression magnitude.

Fragments per kilobase of exon per million fragments mapped (FPKM) calculation: We calculated the number of fragments per kilobase of non-overlapped exon per million fragments mapped (FPKM). Since the raw count data per gene were generated with the “union” mode in HTSeq, where the reads mapped to the overlapping regions between mRNAs and lncRNAs were not counted, the exon sequences for which overlap between mRNAs and lncRNAs exists were excluded when we calculated the gene lengths for both mRNAs and lncRNAs.

Low expression filtering: To reduce noise, we kept only mRNAs with FPKM equal to or above 1 in at least 10% of the samples for downstream analysis.
