# Supplementary material for: Mutational Profiles Reveal an Aberrant TGF-β-CEA Regulated Pathway in Colon Adenomas
Source: PLoS One. 2016 Apr 21;11(4):e0153933. doi: 10.1371/journal.pone.0153933 (PMC4839765; doi:10.1371/journal.pone.0153933)
Supplement: S1 Table — (DOCX) [file pone.0153933.s006.docx]

| **S1 Table. Mutation frequency of adenoma samples** | | |  |  |  |  |  |
| --- | --- | --- | --- | --- | --- | --- | --- |
| Sample name | Location | Pathology | Size (cm) | Age (years) | Mutations (total No.) | Reference genome (No.) | Mutation frequency (mutations/Mb) |
| **WGS** |  |  |  |  |  |  |  |
| MDA49ad | Distal | Tubular | 2 | 63 | 33 | 3,095,693,981 | 0.010659968 |
| MDA50ad | Distal | Tubular | 2 | 65 | 3361 | 3,095,693,981 | 1.085701630 |
| MDA51ad | Proximal | Sessile serrated | 2 | 64 | 3431 | 3,095,693,981 | 1.108313684 |
| MDA80ad | Proximal | Sessile serrated | 2 | 57 | 12 | 3,095,693,981 | 0.003876352 |
| **WTS** |  |  |  |  |  |  |  |
| MDA1ad | Distal | Tubular | 0.5 | 55 | 28 | 72812856 | 0.384547476 |
| MDA2ad | Proximal | Tubulovillous | 0.4 | 58 | 21 | 72812856 | 0.288410607 |
| MDA3ad | Proximal | Sessile serrated | 0.6 | 61 | 34 | 72812856 | 0.466950507 |
| MDA31ad | Distal | Tubular | 0.5 | 66 | 3 | 72812856 | 0.041201515 |
| MDA27ad | Distal | Tubular | >2 | 69 | 289 | 72812856 | 3.969079307 |
| MDA33ad | Distal | Tubular | 1.2 | 49 | 24 | 72812856 | 0.329612122 |
| MDA34ad | Distal | Tubulovillous | 0.5 | 70 | 122 | 72812856 | 1.675528289 |
